# Challenges and solutions to the implementation of studies within a trial: The experiences of the PROMETHEUS programme

**DOI:** 10.1177/26320843221106949

**Published:** 2022-07-06

**Authors:** Catherine E Arundel, Laura Clark, Elizabeth Coleman, Laura Doherty, Adwoa Parker, David J Torgerson

**Affiliations:** York Trials Unit, Department of Health Sciences, Faculty of Science, 8748 University of York, New York, UK

**Keywords:** Studies within a trial, challenges, solutions, methods and methodology, basic concepts of research, trial efficiency

## Abstract

**Background:**

Effective and efficient conduct of randomised controlled trials (RCTs) ensures accurate, timely results and prevents research waste. There is however limited evidence available to inform the design, conduct and reporting of RCTs. A self-contained, randomised Study Within A Trial (SWAT), embedded within a host RCT or cohort study, offers an opportunity to fill this evidence gap. While SWATs are generally easy to implement, a range of challenges to undertaking SWATs have also been identified, however there is limited detail regarding practical solutions to tackle these.

**Methods:**

Information and observations collected from PROMETHEUS members and participating trials, focusing on SWATs across a wide range of questions and settings, was reviewed to identify the challenges and solutions of delivery of a programme of SWATs.

**Results:**

A range of challenges to undertaking SWATs (e.g., obtaining governance approvals) were identified along with potential solutions to these, which were implemented accordingly during programme delivery. Central to the solutions to resolve SWAT challenges is education to develop knowledge and understanding in the wider research community on the importance, purpose, and key methodological principles in relation to SWATs. In addition, the sharing of experience, best practice or resources to prevent or help negotiate the barriers to undertaking SWAT evaluation is also recommended.

**Conclusions:**

Potential solutions to the barriers experienced in the design, conduct and implementation of a programme of SWATs have been identified. As more SWATs are completed, this will further develop evidence to support the mitigation or removal of barriers and in doing so this should increase the efficiency of randomised controlled trials.

## Background

Randomised controlled trials (RCTs) are widely accepted as the ‘gold standard’ for the evaluation of effectiveness in healthcare research. However, ineffective or inefficient trial conduct can result in research waste, increased costs, bias and reduced statistical power,^1,2^ resulting in delays in either the adoption of effective treatments or in the withdrawal of ineffective or harmful treatments.

Despite the importance of efficient and effective trial design and conduct, there is limited evidence available to inform the design, conduct and reporting of RCTs.^3^ As a result funders, trialists, participants and the general public cannot always be sure that a trial is being conducted as efficiently and effectively as it could be.^3^ Strategies believed to improve recruitment or retention of participants are routinely used by studies to try to ensure efficient and effective conduct, despite limited evidence to support their effectiveness for example sending newsletters in advance of questionnaire mailings,^4^ use of media advertising,^5^ and alternative PIS formats.^6^

Studies within a trial (SWATs) offer an opportunity to fill this evidence gap. As self-contained randomised or non-randomised studies, embedded within a host RCT, SWATs aim to evaluate or explore alternative methodologies around trial design, delivery and organisation.^3^ This may include, but is not limited to, strategies associated with participant recruitment,^7,8^ participant retention^9,10^ or other strategies routinely used in the conduct of trials but without evidence of effect.^11,12^ SWATs often evaluate strategies within single host trials.^9,13–15^ To account for the lack of statistical power often observed in individual embedded studies, these require combining in a meta-analysis once data is available from sufficient, similar SWATs. Undertaking evaluation in this way means it can take many years to reach a consensus on effectiveness.

The completion of SWATs has been increasing over time; Cochrane systematic reviews noted 45 eligible recruitment studies in 2010^16^ and 38 eligible retention studies in 2013,^17^ rising to 68 recruitment studies in 2018^18^ and to 71 retention studies in 2020.^19^ This is likely due to the promotion of SWATs by groups such as MRC-START,^20,21^ Trial Forge^22^ and the introduction in April 2018 of a funding component for SWATs within the UK National Institute for Health Research (NIHR) funding application process.^23^ Another important element in increasing the number of SWATs completed has been the Promoting The Use of SWATs (PROMETHEUS) programme, funded by the UK Medical Research Council (MRC)^24^ This programme was conceived to help to increase the conduct of SWATs, and to build the evidence base around effective strategies for use in RCTs; 42 randomised recruitment and retention SWATs within 31 trials have been pump primed by this programme.^25^

SWATs are generally easy to implement within an RCT,^26^ and Trial Forge Guidance 1^3^ outlines key elements which should be considered when planning a SWAT; cost, randomisation, ethics, analysis, implementation, and publication. Despite the general ease of implementation, however, a range of challenges to undertaking SWATs have also been reported previously.^26–29^ These correspond to the key elements identified by the Trial Forge Guidance 1,^3^ with key themes including the impact on the host trial and its participants, resistance from the wider study team, and receipt of study approvals. Despite the identification of challenges, there islimited detail provided on practical solutions to tackle these difficulties in advance of or while undertaking a SWAT evaluation.

Given the number of SWATs undertaken in the PROMETHEUS programme, and the diversity of host trials, patient populations and designs included, it has been possible to further identify and explore challenges experienced when undertaking randomised recruitment and retention SWATs across a wide range of contexts. This has allowed identification and implementation of potential solutions accordingly. This paper describes the challenges to undertaking SWATs, as observed in the PROMETHEUS programme, and offers practical solutions, which may be helpful to trialists when undertaking similar evaluations in the future. This should help to ensure continued and successful conduct of SWATs across the research community.

## Challenges and solutions

### Funding

#### Challenges with studies within a trial funding

The costs of including a SWAT can range widely from less than £100 to over £10,000, and so this may also be seen as a barrier to inclusion in a host trial.^30^ The tight financial constraints of publicly funded research, and the requirements to monitor these, may mean that trials teams are reluctant to invest in SWATs.

#### Solutions to the challenges of studies within a trial funding

When considering SWAT funding, it is important to note that SWAT interventions lie on a continuum in terms of complexity and cost. At one end, inclusion of a SWAT will fit seamlessly with the existing trial, for example, where postal questionnaire mailings are undertaken the direct cost of randomising patients to receive one of two cover letters would be neutral, there should be no difficulty in including this SWAT. To the converse, more complicated SWATs, for example a recruitment training course, will likely incur significant cost and time implications and therefore these may be more difficult to implement in an existing tria. Testing of these may be better suited to being included in targeted SWATs funding, for example via the NIHR funding application process. In the middle are relatively low cost interventions with potential for significant returns, for example inclusion of pens with recruitment materials or follow up questionnaires.^31^ Irrespective of direct costs, all SWATs carry additional indirect costs for example additional randomisation, database changes and analysis time. Where a SWAT is implemented from the outset of a trial, and is analysed alongside the main trial the additional indirect costs can be limited. Trial teams should therefore be encouraged to ‘trade off’ the small additional cost with the potential return in recruitment or retention by including a SWAT accordingly.

### Approvals

A SWAT is designed to be embedded within a host trial with no implication on its integrity, rationale or outcomes., Theoretically it should be relatively simple to obtain governance approvals, however difficulties and the long timeline in obtaining research governance approvals for multicentre research studies has previously been reported in the literature^32–34^ with these difficulties also prevailling in the approvals process for SWATs.

#### Challenges to obtaining studies within a trial approvals

Participating trials noted that queries regarding the SWAT were often raised during the approvals process. Teams noted that they believed this was due to be a lack of knowledge or understanding about the importance, purpose or methodology of a SWAT. Queries were raised by a range of groups including Sponsors, governance bodies (in the UK the Health Research Authority (HRA) and Research Ethics Committees (REC)), oversight committees and patient advisory groups.

A key element of SWAT education centres on the need, or not, for patient consent or information provision in relation to the SWAT. The PROMETHEUS programme was frequently asked to provide support to host trial teams to explain to relevant governance bodies why patient consent and provision of information for the SWAT is unnecessary.^30^ For both recruitment and retention SWATs, provision of any information or a requirement for consent may jeopardise and dilute the SWAT evaluation through the Hawthorne effect and/or resentful demoralisation. In addition, where a SWAT is assessing methods of recruitment approach, given their very nature, it may also be impossible to provide information on or to seek consent for SWAT involvement.

#### Solutions to challenges in obtaining studies within a trial approvals

While education may take time to implement, given the numbers of Sponsors and ethics committees research teams can assist with this effort at an individual study level. Applications including a SWAT should document explicitly what will happen and when this will occur, identifying which elements comprise the host trial and which are part of the SWAT, and who will undertake the SWAT activity, particularly noting if the SWAT is to be undertaken centrally thus limiting any additional actions required by participating sites. Applications should also reflect on the lack of current evidence in relation to recruitment and/or retention, the importance of building this evidence and the opportunity that the study affords to contribute to research efficiency.

To tackle the issue with regards need for patient consent and where additional consent or information is not appropriate, such as for the reasons as noted above, this should be clearly justified in the associated application. It may also be helpful to note the set precedent for the same or similar SWATs, noting other studies where the same SWAT has successfully been approved and embedded. In addition, it may be useful to refer to a SWAT as ‘embedded’ or ‘nested’ rather than as a ‘sub study’; to some ‘sub study’ may infer that additional consent is required.

SWATs will usually require research governance approvals prior to implementation,^3^ usually from the host institution and the national governance or ethics bodies. On occasion however national ethical approval may not be required. A multi trial SWAT, conducted by the PROMETHEUS programme in conjunction with the University of Bristol, sought to test the effectiveness of a recruitment training workshop for site staff on patient recruitment in participating host trials.^35^ This SWAT was deemed by the UK Health Research Authority not to require approval, as this was a staff only intervention being delivered outside of National Health Service (NHS) hospital premises. This decision was reached following consultation with the HRA therefore for similar studies, or where requirement for approval is unclear, this should be discussed with the relevant governance bodies accordingly to ensure a final, confirmed decision is made for the individual study.

Depending on the SWAT intervention, it may be appropriate to undertake an overarching governance application to avoid the need for amendments to be made by each individual trial who undertakes this. The PROMETHEUS programme has successfully used this across eight trials in a Christmas Card SWAT^36^ conducted in 2019. Through this SWAT, it was identified that SWAT components do not always fit into the UK national ethics form ‘Integrated Research Application System’ (IRAS). For example, as noted above, the provision of informed consent and information will not necessarily be appropriate or necessary, however such sections of the IRAS form do afford an appropriate opportunity to justify, in detail, why this is the case.

Depending on whether a SWAT is to be conducted in a single or multiple trials will dictate Sponsorship arrangements. In an individual host trial setting, it is recommended that the Sponsorship of the host trial should also cover the SWAT, however where a concurrent SWAT evaluation is to be undertaken across multiple host trials, it may be preferable for the coordinating institution to cover the Sponsorship for the SWAT. Where evaluation is to be conducted across multiple trials, Sponsors may also need to consider provision of funding, data sharing or collaboration agreements accordingly.

### Studies within a trial implementation

Overall, the reception to SWATs has largely been positive, however occasionally some trial teams have been resistant to their implementation, with the underlying driver appearing to be a lack of understanding and/or appreciation of the importance of SWATs.

#### Challenges to studies within a trial implementation

Given the key focus of trialists and clinicians is to complete the planned RCT to time and to target, an unfamiliarity or lack of understanding of SWAT methodology, may result a misconceived perception that a SWAT adds complexity to a trial and may distract the trial team away from the main study. In addition, where established strategies are proposed as the SWAT intervention, anecdotal evidence can result in a perception that interventions are already effective, and so testing in a SWAT is unnecessary. Where novel strategies are proposed, study teams may wish to implement components of the planned SWAT intervention (wholly or in part) across the study, without randomisation. Much like in clinical trials, introducing interventions without supporting evidence can result in ineffective interventions continuing to be used, or delays in implementing effective strategies, which in turn contributes to research waste.

Generally, SWATs are designed to have limited burden on patients, for example the provision of a reminder letter, pre-notification that a questionnaire will soon arrive, or inclusion of an incentive requires little involvement from the participant. This is not however always the case for study teams, and resistance to include a SWAT may prevail because of perceived lack of time, and/or resources, to facilitate this methodological work.

When the host trial includes a very specific patient population or associated research community, there may also be some concern about the potential for negative impact or demoralisation. Furthermore, during the PROMETHEUS programme concerns were also raised with regards the potential for SWAT interventions and host trial interventions to interact. Interaction is extremely unlikely in the majority of instances; however, the impact of this may differ depending on the study population, trial intervention and/or SWAT intervention

#### Solutions to the challenges of studies within a trial implementation

Implementation of the SWAT should be considered from the design stage. For example in a trial where participants may come into contact with each other and have opportunity to discuss the SWAT intervention, care should be taken during the SWAT design and planning phases to consider appropriate interventions or randomisation methods to try to minimise demoralisation as far as is possible, which could include cluster randomisation.

Consideration of the timing of intervention implementation is also important; for example, it may be more amenable to a trial team to delay introduction of a SWAT to allow core trial activity to stabilise or embed. Considering some level of flexibility with the SWAT design or intervention, while maintaining the core methodological assessment, may also be helpful in mitigating concerns with regards implementation.

The impact of the introduction of a SWAT particularly for study teams, and study participants if applicable, should be made clear from the outset, with attention paid to identifying potential options to reduce this if possible. Availability of resources such as text for inclusion in ethical approvals and intervention templates (available from groups such as PROMETHEUS and Trial Forge) can help to limit the initial set up activity, as can centralisation of SWAT activity to the coordinating centre, if possible. Ultimately, the less additional effort required by the study team (and participants) then perhaps the more likely the SWAT will be implemented.

Adherence to an intervention should be monitored throughout trial delivery, and the same is true for SWAT interventions. In many cases interventions are provided physically (e.g., providing different consent materials as a recruitment strategy or mailing a newsletter as a retention strategy), and increasingly technology is being used to deliver SWAT interventions (e.g., multimedia recruitment interventions or text messaging). Where technology is used, and particularly if this is automated or outsourced to a third party, it is imperative to undertake routine, planned assessment and monitoring, to ensure that the intervention is implemented as planned. This should be planned and introduced from the outset, rather than being reactive to any technological failure.

Alongside practical solutions is education, both in terms of SWAT methodology and of the importance of developing the current evidence base to ensure efficient and effective conduct of future trials. The presence of initiatives such as PROMETHEUS,^25^ Trial Forge^22^ and the NIHR SWATs^23^ funding should help to raise the profile of and educate about the importance of SWATs.

### Publication

Prompt publication of SWAT findings is crucial to enable a meta-analysis to be undertaken to provide a statistically powered assessment of the evidence for a particular recruitment or retention strategy, thus expanding the current, limited evidence base. Despite the importance of this, publication of SWATs in peer-reviewed journals can be difficult.

#### Challenges with studies within a trial publication

There are limited methodological journals that routinely publish SWATs; as a result, such journals may be fatigued given the increases in SWAT activity. Population or condition specific journals offer an alternative; however, by the nature of their focus, there may be limited interest and acceptance of SWAT publications in these journals.

Studies may wish to combine the SWAT findings with the main trial publication; however, this could result in delays in publishing SWAT findings particularly if the SWAT focused on recruitment interventions and the main trial has a long follow up period. A combined publication also does not offer the opportunity for development of publication profiles, particularly for early career researchers, for whom a separate SWAT publication may enable them to establish an authorship presence. Furthermore, combining SWAT findings within a main trial publication may limit future thorough assessment of the evidence base, given that such publications may not appear in a systematic review search strategy focusing specifically on SWAT methodology.

Given the need for SWATs to be combined in a meta-analysis, due to limited statistical power in the individual SWATs, one particular difficulty often identified during publication review is the limited statistical power and/or absence of a formal sample size calculation. As individual SWATs are often underpowered, they may individually show a lack of or limited effect. Including a power calculation therefore runs the risk of discouraging further assessment of such an intervention.

#### Solutions to the challenges of studies within a trial publication

Given the limited number of methodological journals available, the use of publication platforms or appropriate journals which do not mandate article processing fees, may be an appropriate alternative, given they enable prompt, independent publication of a SWAT for a limited cost, comparative to other peer reviewed journals. Within publication platforms, costs are often derived based on publication word count; a succinct SWAT publication therefore has the added benefit of reduced cost and prompt publication – as well as putting less burden on the trial team when writing up the results. The PROMETHEUS team have developed 1000 and 1500 word SWAT reporting templates for this very purpose, and this has already been used successfully.^37–40^

As SWAT findings often need to be combined to ensure sufficient statistical power, it may be prudent to explain in a publication why a sample size calculation has not been included and to note why publication to enable combination via meta-analysis is so important.^3^

Given the limited awareness and education around this methodology, considering peer reviewer nominations is also important to ensure a fair and appropriate review.^30^ It may therefore be advisable to review the available literature to identify and propose reviewers with experience of similar SWATs given they are perhaps more likely to be amenable to the intricacies of SWAT design, methods and reporting.

## Discussion

### Summary of key findings

This article explores the challenges and potential solutions to undertaking SWATs, in the context of the PROMETHEUS programme.

Conducting SWATs remains important to minimise research waste and to maximise research efficiency. Completion of SWATs has developed over time as can be seen by the increase in the number of trials included in Cochrane Methodology reviews. This is attributable to the involvement of groups such as MRC START,^20,21^ Trial Forge,^22^ the MRC funded PROMETHEUS programme,^25^ and the introduction of a funding component for SWATs within the UK NIHR funding application process.^23^

During the conduct of the PROMETHEUS programme, four key themes were identified in relation to challenges experienced in the design, conduct and implementation of SWATs: funding, approvals, implementation, and publication.

For each of the challenges identified in the four themes, the prevailing solution to each was education of the wider research community on the importance, purpose, and key methodological principles in relation to this research design. There are of course policy- and strategic-level discussions that are required here, however individual trialists can also play their part. If trialists continue to undertake SWATs and provide robust justifications and descriptions when seeking necessary regulatory approvals, this will undoubtedly develop knowledge and understanding of this methodology. In addition, the sharing of experience, best practice or resources to help others to prevent or negotiate the barriers to undertaking SWAT evaluation is also recommended.

### Comparison with existing literature

The challenges identified here correspond to those outlined in previous reports.^3,26–29^ Of the six key areas for consideration when implementing a SWAT noted in the Trial Forge Guidance 1^3^ challenges have been identified here in relation to funding, implementation, ethics and publication. Trial Forge Guidance 1 however does not allude to the potential challenges associated with these and does not offer direct solutions to any barriers observed.

Adamson et al.^26^ however have noted many similar challenges to those identified here and provide suggestions on potential solutions, which correlates with many of the strategies identified here. For example, Adamson et al. note cost and sample size as being barriers which were mitigated by undertaking inexpensive interventions and using multiple replications and meta-analyses. Similarly, the review of challenges by Martin-Kerry et al.^28^ identifies many similar challenges to both this work and that of Adamson et al.^26^ An important difference is the challenge of funding, for which Martin-Kerry et al.^28^ suggest additional funding and recruitment accruals for SWATs the UK Clinical Research Network. While this differs to the findings of the PROMETHEUS programme, in which overarching funding was noted as a key barrier, this is another important consideration which may help to support SWAT work accordingly.

Qualitative work by Rick et al.^29^ primarily focussed on the challenges of implementing SWATs with the findings correlating with this current work. The solutions offered for addressing the identified challenges are limited to clarification on available funding to support SWATs and support from the research community, with no practical suggestions on how this may be achieved. Earlier qualitative work by Graffy et al.^27^ with principal investigators, trial managers, funders and ethics committees, however provides evidence to support the recommendations made here that the burden of undertaking SWATs should be minimised as far as possible, by ensuring that the interventions and design are compatible with the host trial.

The key themes identified in this current work largely correlate to earlier similar work in this area. Some suggestions for practical solutions to mitigate or minimise barriers have been provided previously, however this information is often limited, focusing on one specific theme rather than the range identified. This paper therefore further builds on previous work by providing a broad summary of both challenges and solutions.

### Strengths and limitations

This research has been conducted within the largest known programme of work specifically focussing on the undertaking of SWATs in randomised controlled trials. Given the number of SWATs undertaken in the PROMETHEUS programme, diversity of host trials, patient populations and designs included, this has afforded a comprehensive review of challenges and solutions as observed by the trials involved in this programme.

The PROMETHEUS programme was conducted with the UK. While many of the challenges and solutions identified will likely be applicable to SWATs conducted in other countries, it is important to acknowledge that the challenges and solutions experienced in other countries may differ to those outlined. In addition, the PROMETHEUS programme focussed specifically on randomised SWATs of recruitment and retention interventions. Again there may be different challenges and solutions when undertaking non-randomised SWATs or SWATs focussing on other elements of research conduct and delivery. The challenges identified through the PROMETHEUS programme are not conclusive, and individual teams may not account these when undertaking a SWAT or may find other challenges when embedding methodological work within their RCT.

### Implications for future practice

This paper offers practical solutions which may be helpful to trialists when undertaking SWAT evaluations in the future. The continued undertaking of SWATs and sharing of experiences is crucial to increasing knowledge and understanding of this methodology. Best practice and resources will further help to develop a range of strategies to minimise or mitigate further barriers to SWAT evaluation.

## Conclusion

There are barriers to the design, conduct and implementation of SWATs and their findings. We have explored these barriers, along with strategies to address these in the future. The more SWATs are completed, the more routine they will be. Routine completion ofSWATs will increase theevidence base to support the mitigation or removal of barriers. With the removal of such barriers, the more efficient randomised controlled trials can and will become.
